# The Relationship between the US Economy’s Information Processing and Absorption Ratios: Systematic vs Systemic Risk †

**DOI:** 10.3390/e20090662

**Published:** 2018-09-02

**Authors:** Edgar Parker

**Affiliations:** New York Life Insurance Company, 51 Madison Avenue, New York, NY 10010, USA; edgar_parker@newyorklife.com; Tel.: +1-254-258-0675

**Keywords:** systemic risk, systematic risk, portfolio management, absorption ratio, information processing ratio, Shannon entropy, entropic yield curve, regime shift, Markov switching models

## Abstract

After the 2008 financial collapse, the now popular measure of implied systemic risk called the absorption ratio was introduced. This statistic measures how closely the economy’s markets are coupled. The more closely financial markets are coupled the more susceptible they are to systemic collapse. A new alternative measure of financial market health, the implied information processing ratio or entropic efficiency of the economy, was derived using concepts from information theory. This new entropic measure can also be useful in predicting economic downturns and measuring systematic risk. In the current work, the relationship between these two ratios and types of risks are explored. Potential methods of the joint use of these different measures to optimally reduce systemic and systematic risk are introduced.

## 1. Introduction

As demonstrated in the 2008 financial crisis, traditional allocation methods can result in large portfolio value losses from unanticipated economic shocks. This fact has motivated a large increase in interest in measures of systemic risk. The hope is that a such a measure or combination of measures could help anticipate periods of outsized financial risk and indicate time periods where proactive portfolio rebalancing should be initiated.

Systemic risks arise from failures in financial institutions or intermediaries, which can have negative spillover effects in specific isolated sectors or even the entire economy. These shocks are in contrast to systematic risks that are rooted in the overall financial cycle and can cause large declines such as bear markets. Due to the events of 2008, recent academic literature has explored how systemic risks can be controlled by the use of proper regulatory rules and portfolio adjustments. Systematic risks on the other hand are often considered beyond control or mitigation.

De Bandt and Hartmann 2000 [1] differentiate between two types of systemic risk. The first of the two categories is isolated idiosyncratic risk, which affects only one institution, industry or market. Specific new information may adversely impact a narrow part of the economy or may not be as severe in overall impact.

On the other hand, more broadly defined non-idiosyncratic systemic events will affect many institutions or an entire economy. These larger more powerful events could spread in a contagion-like manner across all institutions and sectors. Additionally, De Bandt and Hartmann 2000 [1] describe how macroeconomic downturns can weaken financial institutions, making then more susceptible to larger collective systemic shocks. Both types of systemic shocks are ultimately a symptom of some anomalous behavior in some sector or sectors of the economy. 

Systematic risks arise as the inevitable result of financial and business cycles. Zigrand 2014 [2] describes systematic risks as arising from non-diversifiable aggregate events such as economy wide technology, output, preference, or monetary shocks. The risks that these shocks generate are not due to the redistribution of wealth but its total reduction throughout the economy. Despite these negative effects, the economy as a whole is functioning as it should. 

An ideal measure or combination of measures would detect both the suddenly arising systemic risks as well as the systematic shocks associated with the longer-term evolution of the financial and business cycles. As explained by the author of [1], differentiating between the different types of risks has important implications for financial regulators as well as portfolio holders.

A few of the new measures of systemic risk introduced in the ensuing years include Mahalanobis Distance [3], the SRISK Index [4], and the Absorption Ratio (AR) [5], among others. Each has its own particular strengths and weaknesses.

The Absorption Ratio (AR) has generated particular attention and use among practitioners. This interest arises from AR’s intuitive interpretation, good fit to empirical data, and usefulness in portfolio rebalancing. As Kritzman [5] showed, the AR was able to anticipate many of the financial downturns of the past cycles. 

Parker [6] utilized information theory to develop the concept of entropic yield curve. From this equation, the implied information processing ratio or entropic efficiency of the economy can be derived. This entropic measure can also be useful in predicting economic downturns. Despite their disparate origins, the AR and R/C share important similarities. These similarities, important differences, and the potential for joint use of these measures will be the focus of this paper.

## 2. Materials and Methods

The Kritzman et al. [5] absorption ratio is calculated as seen in formula 1:

(1) $$AR=\frac{\sum_{i=1}^{n}\sigma_{E_{i}}^{2}}{\sum_{j=1}^{N}\sigma_{A_{j}}^{2}}$$

where,

*AR* = Absorption Ratio
*N* = number of assets
*n* = number of eigenvectors in the numerator of the absorption ratio
$\sigma_{E_{i}}^{2}$ variance of the *i*-th eigenvector
$\sigma_{A_{i}}^{2}$ variance of the *j*-th asset.

Kritzman et al. [5] examined the variance of returns of 51 industries that make up the MSCI index from 1 January 1998 through 31 January 2010. They began by calculating an eigenvector that explains the largest proportion of the assets’ total variance. They then proceeded to calculate another orthogonal eigenvector that explains the next greatest proportion of the assets’ total variance. This process is continued for a total of ten eigenvectors as described in [5]. 

Parker [6] modeled the economy as being based on the communication and the computation of information. Given the focus on communication, the Shannon entropy was combined with results from Ross [7] to derive the entropic yield curve. Parker [6] theorized that “...information whether it is communicated through space as traditionally imagined or through time can be lost, corrupted, or otherwise misprocessed. An economy’s efficiency in processing and communicating information is related to the economy’s interest rates through the following entropic yield curve equation…”:

(2) $$r_{parker}=B_{0}+\frac{\ln\left(\sqrt{t}\right)}{t}\left(1-e^{-C_{1}\left(1-\frac{R}{C}\right)}\right)-\frac{ln\left(\sigma \right)}{t}\left(e^{-C_{1}\left(1-\frac{R}{C}\right)}\right)$$

$$\frac{R}{C}\;\mathrm{is}\;\mathrm{the}\;\mathrm{implied}\;\mathrm{relative}\;\mathrm{information}\;\mathrm{processing}\;\mathrm{rate}$$

where from Burnashev [8]:

(3) $$p=e^{-C_{1}\left(1-\frac{R}{C}\right)}$$

where *t* represents the term to expiration, $B_{0}$ is the asymptotic long rate such as the 30-year bond yield, *R* is the current economic information set, and *C* is the economy’s information processing capacity. The remaining terms *C_1_* and *σ* are adjustment constants as described in Burnashev [8] and Parker [6], respectively. Burnashev’s error exponent *p* determines the relative influence of the true distribution compared to the error distribution in the entropic yield curve, as explained in Parker [6].

The Implied Information Processing Rate (IIPR) or R/C can be estimated by matching the entropic yield curve to the observed yields in the markets and then solving for IIPR

**Figure 1:**
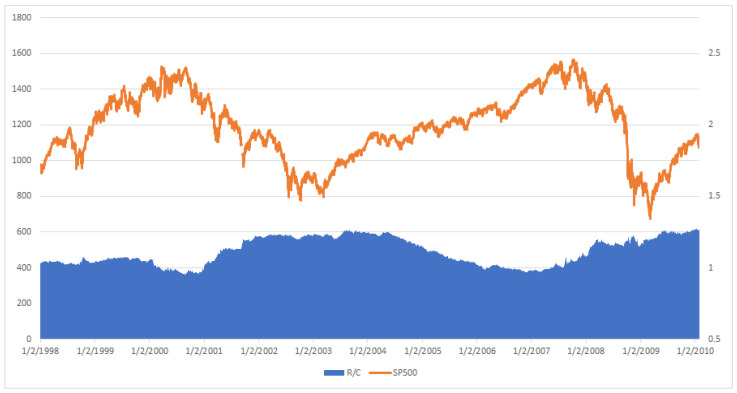
R/C vs. SP500.

The implied information processing rate R/C from 1 January 1998 through 31 January 2010 is plotted against the SP500 index in Figure 1. This can be compared against the absorption ratio (AR) plotted in [5] p 117. (Note the SP500 and MCSI indices are largely composed of the same components and exhibit extremely similar behavior as seen in the figures below. However historical values of the SP500 index are more widely and freely available over longer time periods and are therefore used throughout this paper). Immediately apparent is the similar countercyclical nature of both the AR and R/C against the market measures. The variables AR and R/C seem to rise and fall largely in synchronicity over the two major financial cycles represented in the studied time period. One difference is AR seems to have a trend component in addition to its cycle over the time period, while R/C is trend stationary over its cycle.

## 3. Results

### 3.1. AR Uses and Performance as Described by Kritzman et al.

Kritzman et al. [5] discuss the need to develop tools that can detect systemic risk. The hope is that these proposed tools can help portfolio managers avoid the typical loses during contractions, as returns often become highly correlated during bear markets.

Additionally, Kritzman et al. [5] noted the occurrence of large regime changes between low volatility expansions and high volatility financial downturns over the financial cycles.

Kritzman et al. found:

“…1. Most significant U.S. stock market drawdowns were preceded by spikes in the absorption ratio.

2. Stock prices, on average, depreciated significantly following spikes in the absorption ratio and, on average, appreciated significantly in the wake of sharp declines in the absorption ratio.

3. The absorption ratio was a leading indicator of the U.S. housing market bubble.

4. The absorption ratio systematically rose in advance of market turbulence.

5. Important milestones throughout the global financial crisis coincided with shifts in the absorption ratio…”.

Kritzman et al. [5], p. 113

In the following discussion, an analysis of R/C will produce similar results as Kritzman found for AR. In addition, it will be shown that R/C provides additional information about the complete financial cycle not available with AR. Ultimately the different perspectives and strengths of these two measures could be combined to reduce systemic and regime change induced risk. 

### 3.2. R/C and Polycyclic Portfolio Rebalancing

Systematic shocks have different effects on two the broad classes of assets dubbed procyclical and counter cyclical assets. Procyclical assets tend to rise in value when the economy is expanding and fall with the advent of a recession. Countercyclical assets are instead negatively correlated with the state of the economy. Despite the use of optimization methods, hedging, and *ad hoc* rebalancing techniques, most portfolios are procyclical in behavior and tend to lose value as the economy slows and equity markets fall due to systematic shocks. In this paper, a new portfolio rebalancing technique is introduced, which has the potential to favorably and systematically pivot a portfolio between pro and counter cyclical strategies before regime changes in the economy. This “polycyclic” approach offers potential portfolio returns well above those of traditional methods. This technique can be implemented independently or in conjunction with other portfolio optimization methods. (To be truly polycyclic, the R/C rebalancing would be between long and short positions and/or procyclical and countercyclical assets.)

Traditional strategic asset allocation methods can result in large portfolio value losses from the inevitable changes in economic conditions over time. To better deal with these challenges, regime-based strategies attempt to adjust the portfolio’s risk profile to the current state of the economy. In regime-based asset allocation, the evolution of the economy is described by typically 2–4 regimes that are detected by means of hidden markov models. Regime-based models have been shown to have superior out-of-sample profitability when compared to more rigid investment structures. This article introduces the concept of dynamic asset allocation and portfolio rebalancing via an economy’s relative information processing ratio. This new information theory-based investment method offers advantages over both traditional and regime-based asset allocation methods.

#### Theoretical and Practical Limitations of Traditional Regime Based Strategies

One of the most popular regime models in current use are hidden markov switching models (HMSM). These methods assume that only the current data set is useful in predicting the current state of the economy. In contrast, R/C makes use of the current and past evolution of the economy as evidenced by R/C to determine the current economic state.

Determining the proper number of regimes to include in (HMSM) models is an important but unsolved problem, as per Kasashara and Shimotsu [9]. Too few or too many regimes and the data series cannot be properly modeled; Cavicchioli [10]. “In practice the state dimension of the Markov chain is sometimes dictated by the actual application or it is determined in an informal manner…” Cavicchioli [10]. More formal mathematical methods such as the likelihood ratio test statistic fail for reasons such as “unidentifiable parameters, the true parameter being on the boundary of the parameter space, and the degeneracy of the Fisher information matrix…” Kasashara and Shimotsu [9]. Additionally, there is no explicit justification to assume that the number of regimes is constant as the data series evolves.

### 3.3. The R/C Model and Polycyclic Portfolio Rebalancing (PPR)

Polycyclic Portfolio Rebalancing (PPR) was developed based on ideas from information theory. Parker [6,11] started with the assumption that a hypothetical variable R indicates the amount of information that the economy as a whole is attempting to process and that C is the capacity of the economy to process that information. The ratio R/C is then defined as the economy’s relative information processing ratio. Using an alternative derivation of the yield curve, Parker [6] demonstrated how to derive an estimate of R/C using actual daily yield rates. This allows one to quickly derive a valuable unlagged snapshot of the state of the economy each day at the close of business.

**Figure 2:**
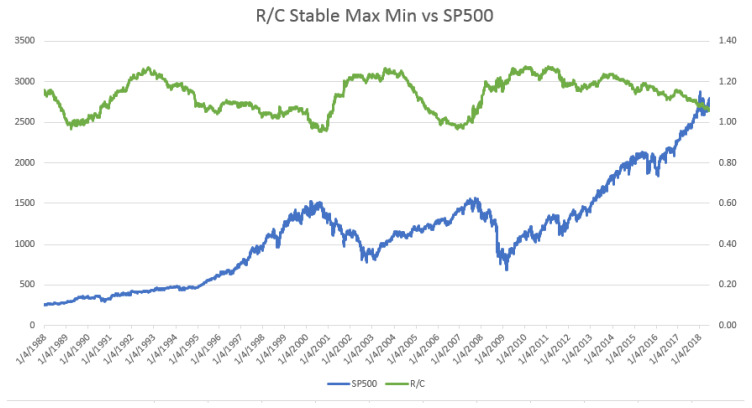
R/C’s Stable Maximum and Minimum vs. SP500 (1988–2018).

Parker [11] examined the time evolution of R/C during bull and bear markets. As seen in Figure 2 and in detail in Parker [11], R/C rises, reaches a maximum, and then falls in a cyclical pattern with stable maximum and minimum values. The evolution of this information process provides a new and intuitive explanation of the boom and bust financial cycles, as seen from an information theoretic perspective. Parker argued that this new variable reveals a new causal factor in the evolution of financial and business cycles; see Parker [6,11].

### 3.4. Polycyclic Portfolio Rebalancing (PPR) using R/C and the Variance of R/C over Financial Cycles

One difficulty portfolio managers face is the fact that market contractions and expansions last for irregular time intervals. Additionally, financial market peaks and troughs are also of non-constant magnitude or amplitude over time. Various techniques have largely unsuccessfully attempted to determine financial cycle lengths. Instead of trying to determine the length of a financial cycle, this paper demonstrates how to use the recurring characteristics of the evolution of R/C to indicate periods of a high probability of regime change.

There are a couple of important characteristics of the evolution of R/C over the business cycle. Similar to the equity markets, R/C experiences well-defined long-term upward and downward movements over time. Secondly in contrast to the equity market price cycles, R/C has a stable amplitude (or stable maximum and minimum values over the cycle), as seen in Figure 2. Since the stable turning points of R/C coincide (or slightly precede those of the equity markets) R/C provides a means of measuring the market’s distance from a turning point. 

**Figure 3:**
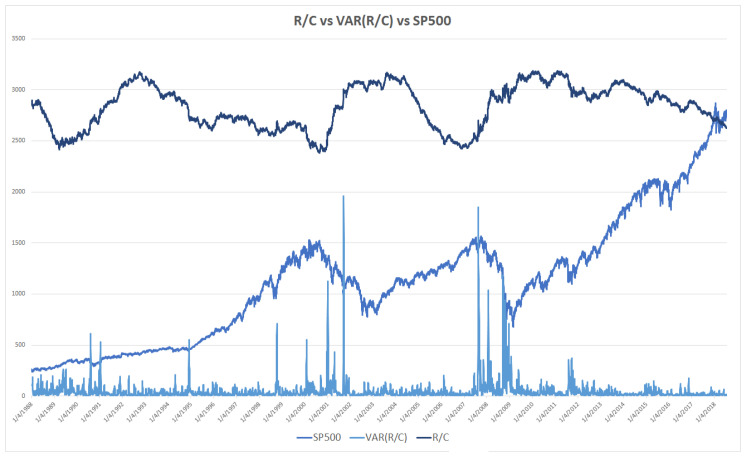
R/C vs. Variance(R/C) vs. SP500 (1988–2018).

Additionally, the combination of different levels of R/C and its variance can be used to provide a range of early warnings of market turns. Parker [6,11] found an inverse relationship between the level of R/C and the variance of R/C. Thus, when R/C nears its minimum, the variance of R/C peaks. The meaning and consequences of the spike in the variance of R/C were explored by Parker [6,11]. Basically, extreme changes in the variance of R/C reflect the explosive loss of information in individual markets and the economy as a whole. The variance of R/C is added to R/C and the SP500 in Figure 3.

**Figure 4:**
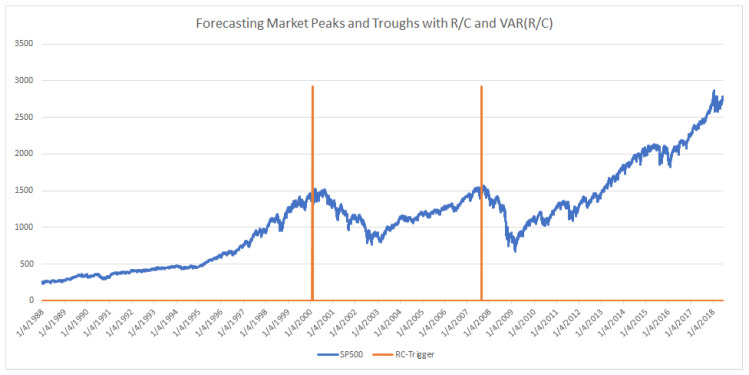
Trigger Level 3: (Bear Market Emergence Signals: 1/4/1988 through 6/18/2018). Activated 50 total days before 3/24/2000 SP500 peak and 43 total days before 10/9/2007 SP500 peak (total days indicates nontrading days included); Logic: R/C <1.02 and VAR(R/C) > 11 times the average variance of R/C in a 10-day window.

When the level of R/C falls to near 1.0 and the variance of R/C spikes dramatically, the probability that the market (SP500 in this case) is near its peak nears 100%. In Figure 4 below, there only two periods that fit the above criteria in over 7600 trading-days of data. Specifically, these data points are generated when R/C <1.02 and VAR(R/C) > 11 times the average variance of R/C in a 10-day window. The indicator alerts below at 50 and 43 days before the 3/24/2000 and 10/9/2007 market peaks, respectively (and nearly as important there are no false alerts over the 7600 trading-day period). (The empirical behavior of R/C and the variance of R/C are consistent with the mathematical theory originally presented in Parker [12]. In that paper, the author hypothesized that when R/C (ratio there is described as CC_A_/CC_L_) approaches 1.00, the behavior of the information processing variable R/C transitions from a normally distributed variable to a Cauchy distributed variable. This would imply that as R/C approaches 1.00, the variance of R/C should also experience a transition to more extreme values.) Just as Kritzman [5] cautioned for AR, R/C triggers should be seen as more of a “near necessary condition for a significant drawdown, just not a sufficient condition”. The R/C measure indicates the high probability and not the certainty of a market decline, despite the impressive performance indicated in Figure 4. 

By adjusting portfolios to take into account the evolving R/C level and variance, the portfolios will automatically be dynamically optimized to the systematic risk generated by the long run evolution of the business cycle. Unlike current methods such as hidden markov models, this new method uses the present day’s data to determine the current state and all past R/C data to indicate the economy’s trajectory along the R/C cycle. Hidden markov models assume that only the last period of data is relevant and only look to determine the current state of the economy and not future evolution.

The portfolio management process is facilitated using rebalancing triggers, which activate at predetermined combinations of the levels and variance of R/C. In the simple example that follows, the triggers can be viewed as long, medium, and short-term indicators of the distance to or probability of a near term market turning point. Portfolios can be adjusted in a systematic and intuitive fashion at the activation of these triggers. 

### 3.5. Simple Example Using the R/C-Cycle Systematic Risk Triggers (Bear Market Emergence)

**Figure 5:**
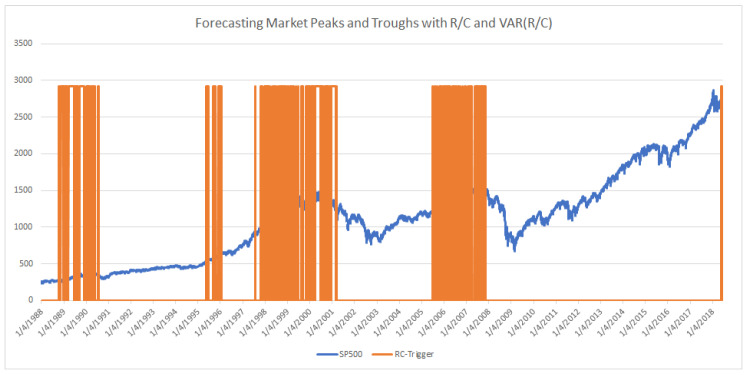
Trigger Level 1: (Bear Market Emergence Signals: 1/4/1988 through 6/18/2018). Began 2 years, 5 months or 882 total days before 3/24/2000 SP500 peak (Includes nontrading days); Began 2 years, 3.5 months or 834 total days before 10/9/2007 SP500 peak (Includes nontrading days); Logic: R/C <1.065 and VAR(R/C) > 0.5 times the average variance of R/C in a 10-day window.

**Figure 6:**
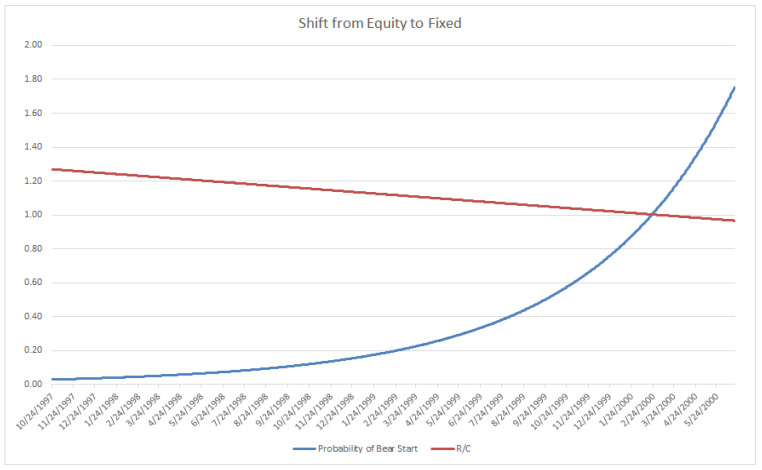
Portfolio Adjustment at Trigger Level 1: Begin exponential increasing shift of portfolio from Equity to Fixed (Most shift occurs in later periods).

One proposed use of triggers for portfolio adjustment is illustrated below, composed of three risk levels to the onset of a bear market (20% or larger market decline). Levels 1, 2, and 3 correspond to long range (very conservative), medium range (less conservative), and shortest range (least conservative) portfolio adjustment criteria, respectively. Note that there are other arrangements and adjustment methods possible, dependent on a particular portfolio manger’s desired risk levels and adjustment criteria. (For example, the current level and rate of change of R/C could be used to determine an estimate of the time till the emergence of a bear market (at R/C ≈ 1.00). This estimated time span could then be inputted into standard probability of loss measures to proactively adjust the portfolio over the R/C cycle. Visit the author’s website www.entropicfinance.com for other such examples.) Once a desired level such as Level 1 has been detected, as seen in Figure 5, long range systematic portfolio adjustment can be initiated. Given that the probability of an immediate downturn is low but increasing, an exponential type of adjustment from equities to fixed may be desired, as demonstrated in Figure 6. Initially the risk of an imminent bear market start is negligible, which would indicate 1% to fixed or other safer investments and 99% to equities. Over time, as R/C falls, the portfolio would increasingly shift to fixed at an increasing rate approaching 100% fixed as R/C reaches 1.00. Note, if the level 1 trigger was not sustained, as seen in Figure 5, the portfolio would be reset to 100% equities awaiting the next trigger alert. The exponentially increasing shift would reduce false trigger loses that turned out not to be sustained long run movements to a bear market. This sort of strategy could be combined with other tactical or systemic risk reduction measures. Interestingly, in the middle of 2018, a level 1 trigger signal was detected using this criterion, as seen at the very end of the sample period in Figure 4. 

Trigger Level 1: (Bear Market Emergence Signals: 1/4/1988 through 6/18/2018).

After trigger begin exponentially increasing shift of portfolio from Equity to Fixed (Most of shift occurs in later periods). 

**Figure 7:**
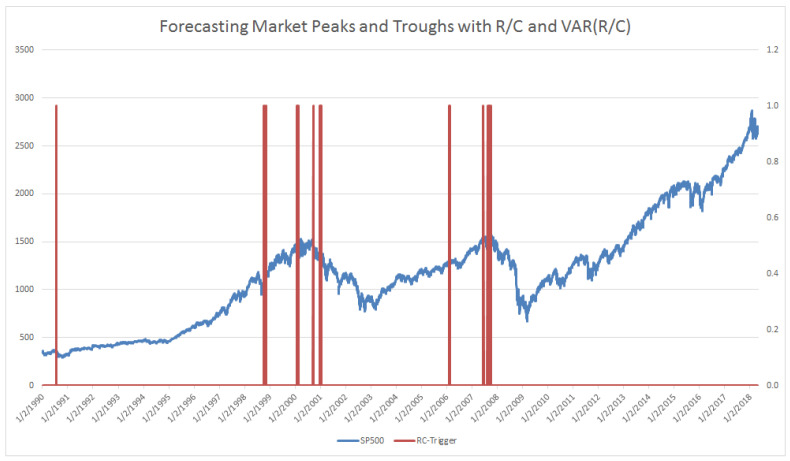
Trigger Level 2: (Bear Market Emergence Signals: 1/4/1988 through 6/18/2018). Began 1 years, 5.3 months or 533 days before 3/24/2000 SP500 peak; Began 1 years, 7.9 months or 603 days before 10/9/2007 SP500 peak; Logic: R/C <1.065 and VAR(R/C) > 4 times the average variance of R/C in a 10-day window.

**Figure 8:**
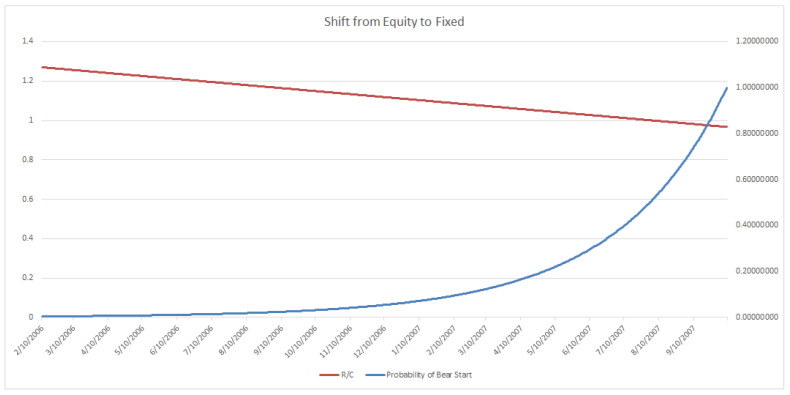
Portfolio Adjustment at Trigger Level 2: Begin exponential increasing shift of portfolio from Equity to Fixed (Most shift occurs in later periods).

A similar reasoning can be used for portfolio managers wishing to utilize the less conservative trigger level 2, which would have activated as seen in Figure 7. The suggested exponential shifting would then have begun, as demonstrated in Figure 8. Finally, when the level 3 riskiest alert, as seen previously in Figure 4, is detected, an immediate shift to 100% fixed or safer portfolio assets is indicated. As mentioned in previously a true “polycyclic” portfolio would take a short position on procyclical assets and or a long position on countercyclical assets at the trigger. Note that this type of R/C-cycle analysis and portfolio adjustment could similarly be done for the emergence of a bull market, as studied by Parker [11].

### 3.6. Summary of the Tradeoffs between the Example Triggers

Trigger Level 1: This is for most risk averse portfolio managers and clients who wish to begin adjusting their portfolio well before the onset of a bear market. This level of user will sacrifice a larger portion of potential gains as the market climbs to its maximum before the onset of a bear market. The user will also have the least risk of suffering losses on missing the onset of a bear market.

Trigger Level 2: This is for less risk averse portfolio managers and clients who wish to begin adjusting their portfolio later than Level 1 users before the onset of a bear market. This level of user will sacrifice less potential gains than a Level 1 user as the market climbs to its maximum before the onset of a bear market. The user will also have less risk of suffering losses on missing the onset of a bear market than Level 3 but more than Level 1.

Trigger Level 3: This is for least risk averse portfolio managers and clients who wish to begin adjusting their portfolio immediately before the onset of a bear market. This level of user will sacrifice a very little of the potential gains as the market climbs to its maximum before the onset of a bear market. This user will also base their turning point on very few days of trigger activation. This level, while accurate over the last 2 cycles over 30 years, also had the fewest trigger points—only 3 activation points beginning 50 days before the 3/24/2000 peak and 1 activation point 43 days before the 10/9/2007 peak. Note that there were zero false positives of these 4 trading days out of over 7600 trading days, and the results conform to the mathematical theory proposed in [12]. In addition to those who wish to shift their portfolio to a conservative stance at the latest possible date as indicated by R/C, this level may be attractive to those who wish to take a large short position before the bear market begins.

## 4. Discussion

### 4.1. AR and R/C Strengths and Weaknesses

Kritzman et al. [5] on page 119 and exhibit 14 discuss using the AR in detecting major economic declines. Specifically, they state that “historic peaks” of the AR preceded the housing bubble related collapse of 2007–2008. But the peaks mentioned occurred over a very wide time span—from years to several months before the actual market peaks and subsequent collapse. Adjusting a portfolio to a conservative stance over such a long period of time would have greatly sacrificed significant market gains. In addition, the “historic peaks” have no logically based level that indicates that one such peak is more or less important than another such peak. One would have difficulty determining if an AR peak, when occurring in real time, indicates that a significant bear market was imminent. Additionally, the absorption ratio studied on page 119 is specifically focused on the housing market. Obviously, one may not know *a priori* which market or sector among many to focus on in the lead up to a recession. Finally, while the AR provides an important early warning of market declines, it also produces false positives. As Kritzman 2011 stated, often “stock prices performed well despite a spike in the absorption ratio” (p. 118). 

As opposed to the AR, the level of R/C in its cycle, combined with the variance of R/C, helps pinpoint when a bear market is likely to emerge. And nearly as importantly R/C can similarly indicate when a shock is not likely to lead to a major bear market decline. In addition, even though different causes are attributed to the bear markets of 2000 and 2007, R/C effectively predicts the downturn starts with no difficulty in determining the significance or timing of the indicator. 

R/C can also be adjusted to the risk level of the portfolio manager or client, as seen with the Level 1, 2, and 3 trigger levels. There is no such mechanism presented by Kritzman et al. [5]. Additionally, R/C does not suffer from the false positives seen in AR at the level 3 trigger level. (Note: the rate of R/C false positives increases as the level of the trigger falls from 3 to 1).

A further difference between the two measures relates to the perspective of the user. For a portfolio manager or client who is focused on a less-interventionist strategy that protects against major bear market declines, R/C is superior. There are fewer false positives, which in themselves reduce potential gains, and fewer costly trades. Alternatively, if a client is comfortable with more frequent interventions that do not necessarily discriminate between minor and major market declines, AR is to be favored. 

One weakness of R/C compared to AR is that R/C is not as effective at detecting the smaller corrections that do not reach the bear market level. This weakness does not reduce the usefulness of R/C’s ability to measure the long-term evolution of the market’s ability to process information. A future avenue of research is to determine if there is an extension of the R/C analysis that can identify these systemic risks.

### 4.2. The Combined use of AR and R/C to Mitigate Systemic and Systematic Risks

R/C and its evolution are driven by the longer-term trends in the informational efficiency of the economy. The AR as currently utilized is more of a shorter-term measure of stability in the financial markets. The stability of the maximum and minimum values of R/C over its long-term cycles adds to its value in reducing portfolio risk. Shorter-term negative and systemic shocks and risks could be mitigated by using AR, while the systematic risk over the longer bull/bear cycles could be more effectively evaluated and responded to by utilizing R/C. 

The cost of confusing the risk of a minor correction versus that of a major bear market drop is obvious. More precise and subtle tuning of the portfolio could be accomplished utilizing both measures. Similarly, AR has no mechanism for indicating the onset of a bull market, or when to fully relax a conservative portfolio stance.

On page 118, Kritzman [5] states that “in many instances, stocks performed well following a spike in the AR.” The author further describes AR as a “near necessary” but not a sufficient condition for a fall in socks. R/C could help refine the use of AR, for as R/C nears its minimum and AR spikes, there is greater evidence that a significant bear market level downturn (20% or more) is near. Or equivalently, a spike in AR when R/C is near its maximum would all but rule out that a bear market was imminent. 

R/C could be thought of as providing a longer-term strategic lens to portfolio management, while AR offers a shorter-term tactical view. Simultaneously attacking both systemic and systematic risks using such objective measures will be of benefit to all portfolio managers and owners. When combined, the AR and R/C ratios reinforce the other measure’s strengths and minimize the weaknesses. 

In addition to very different mathematical and theoretical constructions, AR and R/C are ultimately rooted in different markets. AR is solely derived from equity market data, while R/C is computed from the entropic yield curve equation using only treasury yield data. The use of these variables in concert is of obvious potential value. If more weight is given to the behavior of bond markets over a time period, then R/C should be favored. However, greater reliance on AR would be warranted if the equity markets were deemed to behave more rationally over the same time period. The two measures may reinforce confidence in risk assessment assumptions when in agreement. When in disagreement, the measures may also point to the potential of arbitrage opportunities. 

## Supplementary Materials

Supplementary Materials can be found including the excel file with the macro and data used to estimate R/C at www.entropicfinance.com. The Treasury Yield Curve data is also freely available at: https://www.treasury.gov/resource-center/data-chart-center/Pages/index.aspx.
